# Predictors of inappropriate and excessive use of reliever medications in asthma: a 16-year population-based study

**DOI:** 10.1186/s12890-018-0598-4

**Published:** 2018-02-12

**Authors:** Hamid Tavakoli, J. Mark FitzGerald, Larry D. Lynd, Mohsen Sadatsafavi

**Affiliations:** 1 Faculty of Pharmaceutical Sciences, University of British Columbia, 2405 Wesbrook Mall, Vancouver, BC V6T 1Z3 Canada; 2 Department of Medicine, Institute for Heart and Lung Health, University of British Columbia, Vancouver, Canada; 3 Centre for Clinical Epidemiology and Evaluation, University of British Columbia, Vancouver, Canada

## Abstract

**Background:**

Understanding factors associated with the inappropriate or excessive use of short-acting beta agonists (SABA) can help develop better policies.

**Methods:**

We used British Columbian (BC)‘s administrative health data (1997–2014) to create a retrospective cohort of asthma patients aged between 14 and 55 years. The primary and secondary outcomes were, respectively, inappropriate and excessive use of SABA based on a previously validated definition. Exposures were categorised into groups comprising socio-demographic variables, indicators of type and quality of asthma care, and burden of comorbid conditions.

**Results:**

343,520 individuals (56.3% female, average age 30.5) satisfied the asthma case definition, contributing 2.6 million person-years. 7.3% of person-years were categorised as inappropriate SABA use and 0.9% as excessive use. Several factors were associated with lower likelihood of inappropriate use, including female sex, higher socio-economic status, higher continuity of care, having received pulmonary function test in the previous year, visited a specialist in the previous year, and the use of inhaled corticosteroids in the previous year. An asthma-related outpatient visit to a general practitioner in the previous year was associated with a higher likelihood of inappropriate SABA use. Similar associations were found for excessive SABA use with the exception that visit to respirologist and the use of systemic corticosteroids were associated with increased likelihood of excessive use.

**Conclusions:**

Despite proven safety issues, inappropriate SABA use is still prevalent. Several factors belonging to patients’ characteristics and type/quality of care were associated with inappropriate use of SABAs and can be used to risk-stratify patients for targeted attempts to reduce this preventable cause of adverse asthma outcomes.

**Electronic supplementary material:**

The online version of this article (10.1186/s12890-018-0598-4) contains supplementary material, which is available to authorized users.

## Background

The anti-inflammatory properties of inhaled corticosteroids (ICS) and other asthma controller medications result in sustained improvement in lung function and a reduction in the risk of exacerbations [1]. On the other hand, reliever medications such as short-acting beta agonists (SABAs) are associated with the rapid resolution of symptoms but do not affect the underlying inflammatory process [1]. One of the most important concerns in the treatment of asthma is he adverse effects of the reliever medications, which occurs mostly when the proper balance between the controller and the reliever medication use is not preserved [2]. The evidence strongly suggests that exposure to reliever medications, in the absence of adequate controller therapy, increases airway hyper-responsiveness, which can eventually result in life threatening exacerbations [3–8].

Despite the widespread availability and promotion of guidelines and evidence-based action plans, SABAs continue to be used inappropriately in a large number of individuals [2, 9, 10]. While the outcomes associated with the inappropriate use of SABAs have been studied by many investigators [4, 5, 11, 12], the reasons behind such inappropriate use are unclear.

In a previous study, we have documented a steady decline (5.3% annually) in inappropriate SABA use over a 12-year period in British Columbia (BC), Canada [13]. Such a trend implies that the composition of patients exposed to inappropriate doses of SABAs is rapidly changing over time. In this context, identifying factors associated with inappropriate exposure to SABAs in contemporary patient populations can help narrow the evidence gap and design targeted strategies towards reducing this source of preventable burden. The objective of this study was to evaluate a relatively comprehensive range of factors that could potentially affect the inappropriate or excessive use of SABAs in a population based asthma cohort.

## Methods

We used population-based administrative health data of BC. BC has a universal health-care system covering its entire 4.7 Million (as of 2015 [14]) residents. The administrative needs of such a system have resulted in the creation of centralised databases capturing health-care utilisation records for all of its legal residents. The following databases were available to us: 1-Discharge Abstracts Database (DAD) containing hospitalisation information including admission date and up to 25 discharge diagnoses coded using international classification of diseases, 9th (ICD-9) or 10th (ICD-10) revisions [15], 2-Medical Services Plan (MSP) which contains all outpatient services dates, diagnosis, and costs [16], 3-PharmaNET, which contains dispensation information such as unique drug identifier, service date, dispensed quantities and days of supply, and medication and services costs [17], 4-Vital Statistics database, which contains information on deaths [18], 5-Demographics and Census databases, which contain basic demographic information such as date of birth, sex [19], and census database containing socioeconomic status (income quintiles determined from the geographic neighbourhood) [20]. All data were linkable at the individual level and have shown excellent reliability with very low rate of missing or incorrect data [21].

Data were obtained for the period of January 1st, 1997 through March 31th, 2014. We did not use the first year of data, to allow for sufficient time evaluate the covariates (e.g., comorbidity indices). Hence, the study period was January 1st, 1998 through March 31th, 2014. The Clinical Research Ethics Board at the University of British Columbia approved this study (H15–00062). All inferences, opinions, and conclusions drawn in this research are those of the authors and do not reflect the opinions or policies of the data steward(s).

### Asthma cohort

We created a cohort of individuals with asthma, aged 14 to 55, using a validated and previously applied case definition [22]. According to this definition, asthma was identified if the individual had at least one hospitalisation or used two outpatient services at different dates within a 24-month rolling window. We used the international classification of disease (ICD, 9th revision) codes 493.xx or J45/J46 (ICD, 10th revision) for identifying asthma-specific inpatient and outpatient records.

**Fig. 1:**
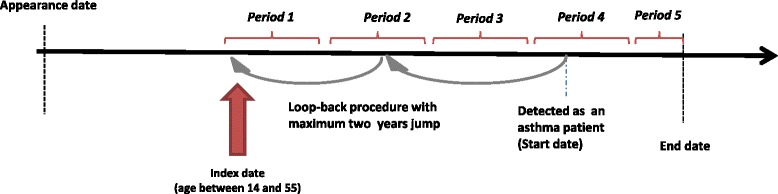
Cohort generation schema

Within this cohort, we applied a ‘look-back’ algorithm to determine the first time the patient used any asthma-related health services. This date was referred to as the index date, marking the beginning of follow-up. Asthma medications were identified using a pre-specified list (Additional file 1). Follow-up continued to the earliest of the following: time of death, end of study period (March, 31, 2014), or the last date of resource use of any type. Follow-up time was divided into adjacent 1 year periods. Figure 1 provides the details of the study design.

### Outcomes and exposures

The primary outcome was inappropriate use of SABAs, as defined previously [23]. Each patient-year of data was labelled as ‘inappropriate use’ if either of the following conditions was satisfied: 1-no use of ICS with 2 or more puffs of SABA per week, or 2-use of more than 9 canisters of SABA during the year and no more than 100 μg/day of ICS [2]. Usage was inferred from the dispensation records. The secondary outcome was excessive use of SABA, defined as filling prescriptions for more than 12 canisters of SABA during the year [24]. The decision to evaluate excessive use independent of inappropriate use was made a priori, as we believe that excessive use can be an independent phenomenon likely to occur in patients with difficult-to-control asthma, despite proper controller therapy.

The exposures were measured during the 12-month period preceding the period in which inappropriate or excessive use was measured (Figure 1). This was conducted to avoid overlapping exposure and outcomes assessment windows, which can cause time-dependent biases. Both controller and reliever medications were adjusted for the defined daily doses (controller medications were adjusted to the beclomethasone equivalent and SABAs to the albuterol equivalent) [4].

### Factors associated with inappropriate or excessive SABA use

We considered three groups of variables for their association with the outcomes: socio-demographic variables, variables pertaining to quality of asthma care, and variables quantifying burden of comorbid conditions. The socio-demographic variables included sex, age, and socio economic status (SES). The latter was defined as income quintiles inferred from geographic neighbourhoods. Age and SES were measured at the beginning of each person-year. Variables pertaining to the quality of asthma care comprised of the receipt of care for asthma by general practitioners, specialist consultations, continuity of care (COC), and whether pulmonary function tests (PFT) was performed. For COC, we calculated the Bice-Boxeman index for each patient-year [25]. This index varies between 0 and 1, with zero meaning that an individual’s physician visits were all to different physicians during the year, and 1 meaning that the individual only consulted with the same physician during the year. The other factors in the category of quality of asthma care were the number of asthma-related hospitalisations, use of any systemic corticosteroids, and appropriate controller medication use. The latter was defined as the ratio of a ICS (either in a single inhaler or a combination inhaler with long-acting beta-agonists) to all inhaled medications (both measured in number of canisters), as defined and validated previously [26], with a cut-off point of 0.5. Finally, comorbidity-related variables were the number of non-asthma-related hospitalisations, number of non-asthma-related outpatient visits, and the modified Charlson comorbidity score [27] (removing all respiratory related conditions).

### Statistical analysis

SAS Enterprise Guide (Version 6.1, Cary, NC, USA) was used for all analyses. The unit of observation was each patient-year of follow up. Generalised linear models with generalised estimating equations (with a binomial distribution and logit link function, given the binary outcomes) were used to account for the clustered nature of the data (multiple observation units within the same patient). The binary dependent variable indicated inappropriate (primary outcome) or excessive (secondary outcome) SABA use. The aforementioned covariates entered the model as independent variables. All afore-mentioned variables were simultaneously included in the regression model and adjusted for. We excluded the periods in which individuals had no record of any asthma-related resource use (hospitalisation, outpatient visits, or medication dispensation) from the regression analysis, as these periods likely represent dormant asthma. However, in a sensitivity analysis we included such periods and repeated all analyses.

## Results

A total of 343,520 individuals were included in the study. The mean age on the index date was 30.5 (SD = 13.3); 193,992 (56.5%) were female. In total, patients contributed 2,623,065 person-years of data. Of these, 24.3% included periods without asthma-related resource use and were removed from the main analysis. Table 1 provides the baseline characteristics of the study sample and overall distribution of outcome variables. Table 2 illustrates the distribution of the exposure variables.

**Table 1:** Demographic characteristics of the final sample

Variable	Value
Total sample size	343,520
Total person years	2,623,065
Person years with no asthma resource use ^a^	638,075 (24.3%)
Average follow up years (SD)	7.64 (5.3)
Inappropriate use of SABA	190,364 (7.3%)
Excessive use of SABAs	24,017 (0.9%)
Asthma related death	122 (< 0.1%)

*SD* standard deviation

^a^These periods were removed from the main analysis but were investigated in a sensitivity analysis

**Table 2:** Rates and frequencies of exposure during the follow-up time

Variable Group	Variable	Value
Socio-demographic variables	Female; N (%)	193,992 (56.5%)
	Age at index date; mean (SD)	30.5 (13.3)
	Socioeconomic status; N (%)	
	quintile 1	38,501 (11.2%)
	quintile 2	52,581 (15.3%)
	quintile 3	65,695 (19.1%)
	quintile 4	81,610 (23.8%)
	quintile 5	102,534 (29.8%)
	Unknown/missing	2599 (0.8%)
Type and quality of carefor asthma (measured in the previous year)*	Having received pulmonary function test	82,765 (3.2%)
	Respirologist consultation	47,957 (1.8%)
	Internal medicine consultation	28,501 (1.1%)
	Allergist consultation	35,405 (1.3%)
	General Practitioner visits	
	No visit	1,803,958 (68.8%)
	1 visit	452,950 (17.3%)
	2 visits	199,816 (7.6%)
	More than 2 visits	166,341 (6.3%)
	Continuity of care (COC)	
	COC = 0	275,396 (10.5%)
	COC > 0 and COC < 50%	2,065,128 (78.7%)
	COC > =50% and COC < 100%	205,991 (7.9%)
	COC = 100%	76,550 (2.9%)
	History of asthma hospitalisation	9936 (0.4%)
	Ratio of ICS to total asthma medications being more than 50%	776,182 (29.6%)
	Use of systemic corticosteroids	330,381 (12.6%)
Comorbidity (measured in the previous year)	Modified Charlson score (SD)	0.1 (2.0)
	None asthma related outpatient resource use	
	< 5 times outpatient service use	543,505 (20.7%)
	<=5 and > 10 times outpatient service use	615,599 (23.5%)
	<=10 and > 20 times outpatient service use	750,000 (28.6%)
	> 20 times outpatient service use	713,961 (27.2%)
	Non-asthma related hospitalisation	417,864 (15.9%)

*All exposure variables are ascertained in the preceding follow-up period

### Inappropriate use of SABA

In 190,364 (7.3%) patient-years, SABAs were used inappropriately. Table 3 provides the results of the regression analyses on the inappropriate and excessive use of SABAs.

**Table 3:** Factors associated with inappropriate and excessive use of SABA

		Inappropriate use			Excessive use		
Group	Variable	Odds Ratio	95% CI	*P* value	Odds Ratio	95% CI	*P* value
			(Lower, Upper)			(Lower, Upper)	
Socio-demographic	Sex (female = 1)	0.67	0.65–0.68	<.0001	0.50	0.47–0.54	<.0001
	Higher SES	0.97	0.96–0.97	<.0001	0.92	0.91–0.94	<.0001
	Year	0.98	0.98–0.98	<.0001	0.99	0.98–0.99	<.0001
	Age (per 10 years increase)	1.05	1.05–1.06	<.0001	1.36	1.33–1.39	<.0001
Type & quality of care for asthma	Having received pulmonary function test	0.86	0.82–0.89	<.0001	0.90	0.83–0.98	0.0006
	Respirologist consultation	0.70	0.66–0.75	<.0001	1.18	1.07–1.31	<.0001
	Internal medicine consultation	0.69	0.65–0.74	<.0001	1.06	0.95–1.18	0.317
	Allergist consultation	0.48	0.45–0.51	<.0001	0.34	0.28–0.41	<.0001
	General Practitioner visits						
	No visit	–	–	–	–	–	–
	1 visit PY	1.24	1.22–1.26	<.0001	1.58	1.51–1.66	<.0001
	2 visits PY	1.29	1.27–1.32	<.0001	2.46	2.33–2.61	<.0001
	More than 2 visits	1.73	1.69–1.77	<.0001	7.24	6.8–7.71	<.0001
	Continuity of care (COC)						
	COC = 0	–	–	–	–	–	–
	COC > 0 and COC < 50%	0.73	0.71–0.75	<.0001	0.92	0.85–0.99	0.0275
	COC > =50% and COC < 100%	0.77	0.75–0.8	<.0001	0.94	0.85–1.03	0.1953
	COC = 100%	0.82	0.79–0.85	<.0001	0.96	0.84–1.08	0.4703
	Asthma-related hospitalisation	1.46	1.34–1.58	<.0001	1.48	1.33–1.65	<.0001
	Appropriate use of ICS	0.10	0.10–0.11	<.0001	0.09	0.09–0.10	<.0001
	Systemic corticosteroid	0.61	0.60–0.63	<.0001	1.80	1.72–1.90	<.0001
Comorbidity-related variables	Modified Charlson score (SD)	0.99	0.97–1.00	0.1371	0.95	0.91–1.00	0.0201
	None asthma related outpatient resource utilisations						
	< 5 times	–	–	–	–	–	–
	<=5 and > 10 times	0.77	0.76–0.79	<.0001	0.84	0.79–0.89	<.0001
	<=10 and > 20 times	0.68	0.67–0.7	<.0001	0.79	0.74–0.85	<.0001
	> 20 times	0.63	0.61–0.65	<.0001	0.81	0.75–0.88	<.0001
	None asthma related hospitalisation	1.09	1.07–1.11	<.0001	1.33	1.27–1.39	<.0001

Entire covariates have been simultaneously included in the regression model

Among the sociodemographic variables, female sex (odds ratio [OR] = 0.67, 95%CI 0.65–0.68, *P* < 0.001), younger age at baseline (OR = 0.95 per 10-year decrease, 95%CI 0.95–0.96, P < 0.001), and higher SES (OR = 0.97 per one unit increase in quantile, 95%CI 0.96–0.97, *P* < 0.001) were associated with a lower likelihood of inappropriate SABA use. Among type and quality of care metrics, appropriate use of ICS (ratio of ICS of total asthma-related medications being above 0.5) was strongly associated with a lower risk of inappropriate SABA use in the next year (OR = 0.10 95%CI 0.10–0.11, *P* < 0.001).

In a majority (95.1%) of patient-years, general practitioners (GPs) were the sole provider of outpatient care. Receiving at least one asthma-related consultation with a respirologist (OR = 0.70, 95%CI 0.66–0.75, *P* < .0001), internal medicine specialist (OR = 0.69, 95%CI 0.65–0.74, P < .0001), or allergist (OR = 0.48, 95%CI 0.45–0.51, P < .0001), compared with no such consultation, was significantly associated with a lower risk of inappropriate use in the next year. On the other hand, an asthma-related GP visit in a given year was associated with higher risk of inappropriate SABA use in the next patient-year compared with periods with one GP visit. Further, there was an increasing trend between the number of asthma-related GP visits and risk of inappropriate SABA use with the strongest association for patient-years with more than 2 GP visits (OR =1.73, 95%CI 1.69–1.77, *P* < .0001). All levels of continuity of care (COC) were associated with lower risk of inappropriate use compare to person-years in which COC was zero (OR for the highest level of COC [versus COC = 0] = 0.82, 95%CI 0.79–0.85, *P* < .0001).

In the comorbidity indicators category, Charlson score and number of non-asthma-related hospitalisations showed no statistically significant association with inappropriate use of SABA. On the other hand, there was a strong association between the number of non-asthma-related outpatient encounters and lower likelihood of inappropriate use of SABA (OR for person-years with more than 20 visits a year [compared with person-years with less than five visits] = 0.63, 95%CI 0.61–0.65, *P* < .0001). Beside the socio-demographic factors, effect of the rest of exposures belong to preceding year.

### Excessive use of SABA

In 24,017 (0.9%) person-years, SABAs were used excessively (more than 12 canisters per year). Among these, 6840 (28.5%) were also categorised as inappropriate use. In general, the direction of associations included covariates and excessive SABA use was similar to that of inappropriate use (Table 3). The exceptions were the visit of a respirologist (OR =1.18, 95%CI 1.07–1.31, *P* < .0001) and the use of systemic corticosteroids (OR =1.80, 95%CI 1.72–1.90, P < .0001) that were associated with an increased likelihood of excessive use.

### Sensitivity analysis

Additional file 2 illustrates the results for the sensitivity analysis after repeating the analyses based on all person-years including those with no history of asthma-related resource use. The direction and magnitude of the associations for all exposures were similar with those of the main analysis.

## Discussion

We evaluated the association between several patient- and care-related factors and inappropriate/excessive use of SABAs. We found that patients who received appropriate amount of ICS, visited a specialist, or had better continuity of care were less likely to use SABAs inappropriately in the following year. In addition, we found that individuals with a higher SES had a lower likelihood of inappropriate use of SABAs. On the other hand, patients who had more frequent general practitioner visits for asthma had a higher likelihood of inappropriate SABA use in the following year. Overall, many modifiable factors representing type and quality of care (e.g., GP visits, specialist visits, continuity of care, history of PFT) were associated with inappropriate use of SABAs, indicating that inappropriate SABA use is at least partially preventable. An important finding was the strong negative association with previous appropriate ICS use and future excessive SABA use. These results indicate that excessive SABA use, even in severe asthma, can be prevented with adequate controller therapy.

While excessive SABA use was less prevalent than inappropriate use, factors associated with both outcomes were generally similar, with two exceptions: the use of systemic corticosteroids and the visit to a respirologist were associated with a lower likelihood of inappropriate use but a higher likelihood of excessive use. Both findings can be attributable to the residual confounding effect of asthma severity not captured in the other covariates. For example, it is likely that specialists tend to better adhere to respiratory guidelines, as compared with generalists, thus leading to lower levels of inappropriate reliever use. However, patients with very severe or difficult-to-treat asthma, who require high dose reliever therapy, are more likely to be referred to a respirologist, resulting in a higher proportion of excessive SABA use.

Some of the reported associations in the present study have been previously reported. Blanchette et al. demonstrated that patients are more likely to be prescribed an ICS if they have a respirologist or allergy consultation (thus reducing the risk of inappropriate reliever use) [28]. Other investigators have shown that women tend to be more adherent to prescribed asthma therapies than men, which is compatible with their observed the lower risk of inappropriate reliever use in our study [29]. Consultation with a specialist has previously been associated with more appropriate use of asthma medications [30]. To the best of our knowledge, other associations namely the SES and continuity of care have not been previosuly evaluated. There may be many reasons for the low SES being associated with higher rate of inappropriate asthma medication use. In addition to potential differences in environmental risk factors, access to high quality care might be difficult for patients who are socio-economically challenged. In addition, while the public healthcare system in Canada provides free inpatient and outpatient care, medication is generally not covered.Hence lower-income individuals might have difficulties affording the controller medications (e.g., combination inhalers of ICS and LABA). A previous study has illustrated a strong socio-economic gradient in the burden of asthma even in a public health-care system such as that of BC [31].

The major strength of this study was its large, population-based sample with a long follow-up time, which provided estimates of association with a low level of uncertainty. The universal coverage of the health-care system means there was no self-selection through enrolment. Thus our findings can have high external validity in the jurisdictions with similar health-care settings. However, the limitations of the study should also be acknowledged. Filling prescriptions does not equate usage. As such, the associations reported in this study are diluted by the extent medication dispensation deviates from the actual intake. In addition, we could not evaluate several important variables that could moderate the effect of, or interact with, the studied variables (e.g., smoking status, education, levels of airflow obstruction, asthma severity, and patient adherence to medication). Evaluating such associations requires databases with richer clinical content but this will likely come at the cost of generalisability and external validity of the results given the inevitable self-selection of patients into clinical cohorts.

## Conclusions

Considering the high prevalence of asthma, the observed level of inappropriate use of reliever medications results in thousands of patients being at risk of preventable adverse outcomes every year. In a separate work base on the same data, it has been shown that inappropriate SABA use continues to be associated with adverse asthma-related outcomes, specifically a 45% increase in risk of asthma-related hospitalization, 25% increase in asthma-related ED visits, and 6.5% increase in asthma-related medication cost [32]. Our study shows that several factors associated with inappropriate use are potentially modifiable, specifically factors pertaining to the type and quality of care. Indeed, previous research has demonstrated that simple advice to physicians prescribing relievers can result in significant decreases in the total reliever use [33]. Given that the majority of asthma patients in our sample received general practice care, interventions aimed at improving general practitioner’s adherence to evidence-based guidelines have the potential to improve asthma medication use. Future studies need to evaluate the behavioural factors and the role of patient education as potential determinants of appropriate asthma treatment.

## Additional files

Additional file 1: List of asthma-related medications. (DOCX 14 kb)

Additional file 2: Sensitivity analysis after including all patient-years with no history of asthma related healthcare use. (DOCX 18 kb)

## Abbreviations

BC: British Columbia
COC: Continuity of care
DAD: Discharge Abstracts Database
GP: General practitioners
ICD: International classification of diseases
ICS: Inhaled corticosteroids
MSP: Medical Services Plan
OR: Odds ratio
PFT: Pulmonary function test
SABA: Short-acting beta agonists
SES: Socio economic status

## References

[1] Lougheed MD, Lemiere C, Ducharme FM, Licskai C, Dell SD, Rowe BH (2012). Canadian thoracic society 2012 guideline update: diagnosis and management of asthma in preschoolers, children and adults. Can Respir J J Can Thorac Soc.

[2] Anis AH, Lynd LD, Wang XH, King G, Spinelli JJ, FitzGerald M (2001). Double trouble: impact of inappropriate use of asthma medication on the use of health care resources. CMAJ Can Med Assoc J J Assoc Medicale Can.

[3] Taylor DR, Sears MR, Cockcroft DW (1996). The beta-agonist controversy. Med Clin North Am.

[4] Hong SH, Sanders BH, West D (2006). Inappropriate use of inhaled short acting beta-agonists and its association with patient health status. Curr Med Res Opin.

[5] Abramson MJ, Walters J, Walters EH (2003). Adverse effects of beta-agonists: are they clinically relevant?. Am J Respir Med Drugs Devices Interv.

[6] Suissa S, Ernst P, Boivin JF, Horwitz RI, Habbick B, Cockroft D (1994). A cohort analysis of excess mortality in asthma and the use of inhaled beta-agonists. Am J Respir Crit Care Med.

[7] Spitzer WO, Suissa S, Ernst P, Horwitz RI, Habbick B, Cockcroft D (1992). The use of beta-agonists and the risk of death and near death from asthma. N Engl J Med.

[8] Sears MR (1986). Why are deaths from asthma increasing?. Eur J Respir Dis Suppl.

[9] Friedman HS, Eid NS, Crespi S, Wilcox TK, Reardon G (2009). Retrospective claims study of fluticasone propionate/salmeterol fixed-dose combination use as initial asthma controller therapy in children despite guideline recommendations. Clin Ther.

[10] Breton M-C, Lelorier J, Forget A, Blais L (2007). Use of combination therapy in asthma: are they prescribed according to guidelines. Respir Med.

[11] Gerald JK, Carr TF, Wei CY, Holbrook JT, Gerald LB (2015). Albuterol overuse: a marker of psychological distress?. J Allergy Clin Immunol Pract.

[12] Camargo CA, Spooner CH, Rowe BH. Continuous versus intermittent beta-agonists in the treatment of acute asthma. Cochrane Database Syst Rev. 2003:CD001115. https://www.ncbi.nlm.nih.gov/pmc/articles/PMC5650161/. 10.1002/14651858.CD001115 PMC8407022 14583926

[13] Sadatsafavi M, Tavakoli H, Lynd L, FitzGerald JM (2017). Has asthma medication use caught up with the evidence?: a 12-year population-based study of trends. Chest.

[14] Population Estimates,BC Stats,central statistical agency of the Province of British Columbia. 2015.https://www2.gov.bc.ca/gov/content/data/statistics/people-population-community/population/populationestimates. Accessed Feb 2018.

[15] Canadian Institute for Health Information [creator] (2014): Discharge Abstract Database (Hospital Separations). Population Data BC [publisher]. Data Extract. MOH (2014). http://www.popdata.bc.ca/data. Accessed Feb 2018.

[16] British Columbia. Ministry of Health Services MSP fee-for-service payment analysis | BC Government Publication | Legislative Library | Legislative Assembly of British Columbia. https://www.health.gov.bc.ca/msp/paystats/index.html. Accessed 29 Oct 2010.

[17] BC Ministry of Health [creator] (2014): PharmaNet. BC Ministry of Health [publisher]. Data Extract. Data Stewardship Committee (2013). http://www.popdata.bc.ca/data. Accessed Feb 2018.

[18] BC Vital Statistics Agency [creator] (2013): Vital Statistics Deaths. V2. Population Data BC [publisher]. Data Extract. BC Vital Statistics Agency (2013). http://www.popdata.bc.ca/data. Accessed Feb 2018.

[19] BC Vital Statistics Agency [creator] (2013): Vital Statistics Births. Population Data BC [publisher]. Data Extract. BC Vital Statistics Agency (2013). http://www.popdata.bc.ca/data. Accessed Feb 2018.

[20] British Columbia Ministry of Health [creator] (2013): Consolidation File (MSP Registration & Premium Billing). Population Data BC [publisher]. Data Extract. MOH (2014). http://www.popdata.bc.ca/data. Accessed Feb 2018.

[21] Williams J, Adger W (1996). Inventory of studies on the accuracy of Canadian health administrative databases.

[22] Sadatsafavi M, Lynd LD, Fitzgerald JM (2013). Post-hospital syndrome in adults with asthma: a case-crossover study. Allergy Asthma Clin Immunol Off J Can Soc Allergy Clin Immunol.

[23] Lynd L, Guh D, Paré P, Anis A (2002). Patterns of inhaled asthma medication use: a 3-year longitudinal analysis of prescription claims data from British Columbia, Canada. Chest.

[24] Lamb CA, Hoban J, Glaves L, Jahn K, Martin C, Tacinas C, Hay S, Dotts J, Flynn D, Kleerup M, Williams J, McGaw J (2006). Reducing short acting beta agonist overuse in the asthma population. J Allergy Clin Immunol.

[25] Romano MJ, Segal JB, Pollack CE (2015). The association between continuity of care and the overuse of medical procedures. JAMA Intern Med.

[26] Laforest L, Licaj I, Devouassoux G, Chatté G, Belhassen M, Van Ganse E, et al. Relative exposure to controller therapy and asthma exacerbations: a validation study in community pharmacies. Pharmacoepidemiol Drug Saf. 2014;23(9):958–64. 10.1002/pds.3668. 10.1002/pds.3668 24946177

[27] Quan H, Sundararajan V, Halfon P, Fong A, Burnand B, Luthi J-C (2005). Coding algorithms for defining comorbidities in ICD-9-CM and ICD-10 administrative data. Med Care.

[28] Blanchette CM, Culler SD, Ershoff D, Gutierrez B (2009). Association between previous health care use and initiation of inhaled corticosteroid and long-acting beta2-adrenergic agonist combination therapy among US patients with asthma. Clin Ther.

[29] Sundberg R, Torén K, Franklin KA, Gislason T, Omenaas E, Svanes C (2010). Asthma in men and women: treatment adherence, anxiety, and quality of sleep. Respir Med.

[30] Jobin M-S, Moisan J, Bolduc Y, Dorval E, Boulet L-P, Grégoire J-P (2011). Factors associated with the appropriate use of asthma drugs. Can Respir J.

[31] Chen W, Lynd LD, FitzGerald JM, Sadatsafavi M (2016). Influences of socioeconomic status on costs of asthma under universal health coverage. Med Care.

[32] FitzGerald JM, Tavakoli H, Lynd LD, Al Efraij K, Sadatsafavi M (2017). The impact of inappropriate use of short acting beta agonists in asthma. Respir Med.

[33] Wong MD, Manley RT, Stettin G, Chen W, Salmun LM (2010). Intervention to reduce unnecessary dispensing of short-acting {beta}-agonists in patients with asthma. Ann Pharmacother.

