# Supplementary material for: Predictors of inappropriate and excessive use of reliever medications in asthma: a 16-year population-based study
Source: BMC Pulm Med. 2018 Feb 12;18:33. doi: 10.1186/s12890-018-0598-4 (PMC5809893; doi:10.1186/s12890-018-0598-4)
Supplement: Supplementary file 1 — List of asthma-related medications. (DOCX 14 kb) [file 12890_2018_598_MOESM1_ESM.docx]

# Additional file 1: list of asthma-related medications

| ***Asthma Medication Group*** | ***Dins*** |
| --- | --- |
| ***Inhaled Glucocorticoid Steroid*** | ***00374407,00828521,00828548,00851752,00851760,00852074,00872334,00893633,00897353,01949993,01950002,01978918,01978926,02079976,02174731,02174758,02174766,02174774,02213583,02213591,02213605,02213613,02213710,02213729,02215039,02215047,02215055,02216531,02229099,02237244,02237245,02237246,02237247,02242029,02242030,02244291,02244292,02244293,02285606,02285606,02285614,02285614,02303671,02303671*** |
| ***Inhaled corticosteroid and Long-acting beta agonists*** | ***02240835,02240836,02240837,02245126,02245127,02245385,02245386,*** |
| ***Short-Acting Beta Agonists*** | ***00003891,00249920,00620955,00620963,00786616,00790419,00812463,00832758,00832766,00851841,00860808,00867179,00874086,00894249,00894257,00897345,01926934,01932691,01938851,01938878,01945203,01947222,01986864,02022125,02035421,02046741,02048760,02063689,02069571,02084333,02091186,02146843,02146851,02148617,02152568,02154412,02164434,02164442,02165368,02165376,02173360,02192675,02208229,02208237,02208245,02212315,02212323,02212390,02213400,02213419,02213427,02213435,02213443,02213451,02213478,02213486,02214997,02215004,02215616,02215624,02215632,02216949,02229862,02231430,02231488,02231678,02231783,02231784,02232570,02232987,02236783,02236931,02236932,02236933,02239365,02239366,02241497,02243115,02243828,02244914,02245669,02259583,02261324,02326450*** |
| ***Systemic Glucocorticoid Steroid*** | ***00015016,00015024,00016241,00016438,00016446,00016462,00021695,00028096,00028185,00030600,00030619,00030627,00030635,00030643,00030651,00030678,00030759,00030767,00030910,00030929,00030988,00036129,00036137,00036366,00156876,00176834,00210188,00213624,00232378,00252417,00271373,00271381,00280437,00285471,00295094,00312770,00354309,00489158,00508586,00550957,00598194,00607517,00610623,00664227,00716715,00732885,00732893,00751863,00783900,00868426,00868434,00868442,00872520,00872539,00874582,00878618,00878626,00888206,00888214,00888222,00888230,01934325,01934333,01934341,01946897,01964070,01964968,01964976,01977547,02063190,02063697,02063700,02063719,02063727,02194082,02194090,02204266,02204274,02230210,02230211,02231893,02231894,02231895,02232748,02232750,02237044,02237045,02237046,02237835,02239534,02240684,02240685,02240687,02241229,02245400,02245406,02245407,02245408,02250055,02260298,02260301,02261081,02279363,02311267*** |
