# Supplementary material for: Predictors of inappropriate and excessive use of reliever medications in asthma: a 16-year population-based study
Source: BMC Pulm Med. 2018 Feb 12;18:33. doi: 10.1186/s12890-018-0598-4 (PMC5809893; doi:10.1186/s12890-018-0598-4)
Supplement: Supplementary file 2 — Sensitivity analysis after including all patient-years with no history of asthma related healthcare use. (DOCX 18 kb) [file 12890_2018_598_MOESM2_ESM.docx]

Additional file 2: sensitivity analysis after including all patient-years with no history of asthma related healthcare use.

|  | **Inappropriate use** | | **Excessive use** | |
| --- | --- | --- | --- | --- |
| **Variable** | **Odds Ratio (95% CI, Lower, Upper)** | **P value** | **Odds Ratio (95% CI, Lower, Upper)** | **P value** |
|  |  |  |  |  |
| Sex (female=1) | 0.68 (0.67-0.70) | <0.001 | 0.50 (0.47-0.54) | <0.001 |
| Higher SES | 0.97 (0.96-0.97) | <0.001 | 0.92 (0.91-0.94) | <0.001 |
| Year | 0.95 (0.95-0.95) | <0.001 | 0.99 (0.98-0.99) | <0.001 |
| Age (per 10 years increase) | 1.09 (1.08-1.09) | <0.001 | 1.36 (1.33-1.39) | <0.001 |
| Having received pulmonary function test | 0.84 (0.81-0.87) | <0.001 | 0.9 (0.83-0.98) | 0.0125 |
| Respirologist consultation | 0.73 (0.69-0.77) | <0.001 | 1.18 (1.07-1.31) | 0.0015 |
| Internal medicine consultation | 0.74 (0.70-0.79) | <0.001 | 1.06 (0.95-1.18) | 0.3022 |
| Allergist consultation | 0.67 (0.62-0.71) | <0.001 | 0.34 (0.28-0.41) | <0.001 |
| General Practitioner visits |  |  |  |  |
| No visit | - | - | - | - |
| 1 visit PY | 2.29 (2.26-2.32) | <0.001 | 1.58 (1.51-1.66) | <0.001 |
| 2 visits PY | 2.3 (2.26-2.35) | <0.001 | 2.46 (2.33-2.61) | <0.001 |
| More than 2 visits | 2.83 (2.76-2.91) | <0.001 | 7.24 (6.80-7.71) | <0.001 |
| Continuity of care (COC) |  |  |  |  |
| COC=0 | - | - | - | - |
| COC>0 and COC<50% | 0.75 (0.73-0.76) | <0.001 | 0.92 (0.85-0.99) | 0.0275 |
| COC>=50% and COC<100% | 0.79 (0.77-0.81) | <0.001 | 0.94 (0.85-1.03) | 0.1953 |
| COC=100% | 0.82 (0.79-0.85) | <0.001 | 0.96 (0.84-1.08) | 0.4703 |
|  |  |  |  |  |
| Asthma-related hospitalisation | 1.19 (1.11-1.28) | <0.001 | 1.48 (1.33-1.65) | <0.001 |
| Appropriate use of ICS | 0.14 (0.13-0.14) | <0.001 | 0.09 (0.09-0.10) | <0.001 |
| Oral corticosteroid | 0.84 (0.83-0.86) | <0.001 | 1.80 (1.72-1.90) | <0.001 |
| Modified Charlson score (SD) | 0.98 (0.96-0.99) | 0.01 | 0.95 (0.91-0.99) | 0.0437 |
| None asthma related outpatient resource utilizations |  |  |  |  |
| <5 times | - | - | - | - |
| <=5 and >10 times | 0.76 (0.75-0.77) | <0.001 | 0.84 (0.79-0.89) | <0.001 |
| <=10 and >20 times | 0.68 (0.67-0.69) | <0.001 | 0.79 (0.74-0.85) | <0.001 |
| >20 times | 0.67 (0.65-0.68) | <0.001 | 0.81 (0.75-0.88) | <0.001 |
| None asthma related hospitalisation | 1.11 (1.09-1.12) | <0.001 | 1.33 (1.27-1.39) | <0.001 |
